# Enzyme-Responsive Polymeric Drug Delivery Systems for the Treatment of Inflammatory Bowel Diseases: A Review

**DOI:** 10.3390/polym18101146

**Published:** 2026-05-07

**Authors:** Junru Li, Xuanran Yu, Da Huang

**Affiliations:** College of Biological Science and Engineering, Fuzhou University, Fuzhou 350108, China; junruli2026@163.com (J.L.); xuanryu@163.com (X.Y.)

**Keywords:** inflammatory bowel disease, enzyme-responsive drug delivery, colon targeting, nanomedicine, polymer

## Abstract

Inflammatory bowel disease (IBD), including Crohn’s disease and ulcerative colitis, is a chronic inflammatory disorder of the gastrointestinal tract that imposes an increasing global health burden. Conventional pharmacological treatments are often limited by systemic side effects and insufficient drug accumulation at inflamed intestinal sites. Enzyme-responsive polymeric drug delivery systems have emerged as a promising strategy to overcome these limitations by enabling site-specific and controlled drug release within the pathological microenvironment of the colon. This review summarizes recent advances in enzyme-responsive polymeric platforms designed for IBD therapy. We first discuss the altered enzymatic landscape in the intestinal microenvironment of IBD, including host-derived inflammatory enzymes such as esterases, matrix metalloproteinases, and hyaluronidase, as well as microbiota-derived enzymes such as azoreductase, cellulase, and amylase. These enzymes provide intrinsic biological triggers for selective polymer degradation and drug release. We then categorize enzyme-responsive polymeric delivery systems according to the enzymes involved and highlight representative material design strategies, including polymer prodrugs, core–shell nanocarriers, enzyme-degradable hydrogels, and polysaccharide-based carriers. Particular emphasis is placed on the multifunctional roles of polymers that enable targeted delivery, mucosal adhesion, and therapeutic synergy through bioactive degradation products. Finally, current challenges and future directions toward multi-stimuli-responsive systems and clinically translatable polymeric nanomedicine for precision IBD therapy are discussed.

## 1. Introduction

Inflammatory Bowel Disease (IBD), primarily comprising Crohn‘s disease and ulcerative colitis, is understood as a chronic, immune-mediated inflammatory condition of the gastrointestinal tract, in which the host immune response to the gut microbiota plays a central pathogenic role [1]. These two forms are defined as distinct chronic, relapsing disorders characterized by intestinal inflammation and ulceration [2]. Since the 20th century, the global incidence of IBD has continued to rise, evolving from a common condition in Western industrialized nations into a worldwide public health challenge. Epidemiological data indicate that in some countries across North America, Europe, and Oceania, the prevalence of IBD exceeds 0.3%, while case numbers in Asia, Africa, and South America are also increasing rapidly year by year [3,4,5,6]. The increasing burden of the disease makes the development of safer and more effective therapeutic strategies particularly urgent.

Currently, there is no definitive cure for IBD. In clinical practice, immunosuppressive and anti-inflammatory agents, including aminosalicylates, glucocorticoids, and monoclonal antibodies, are widely used for IBD management [7,8]. However, due to the lack of delivery tools capable of precisely transporting drugs to inflamed colonic sites [9,10], the clinical application of many potent therapeutic agents has been severely restricted by their inability to efficiently accumulate and release at the lesion sites. Consequently, current IBD treatments continue to face significant challenges, including substantial systemic side effects and suboptimal patient response rates [11]. These challenges have therefore spurred intensive exploration of biomaterials capable of interacting with disease-related pathological signals at IBD lesions. Among these materials, polymers have emerged as particularly promising candidates due to their tunable chemical structures and excellent biocompatibility [9,10].

**Figure 1:**
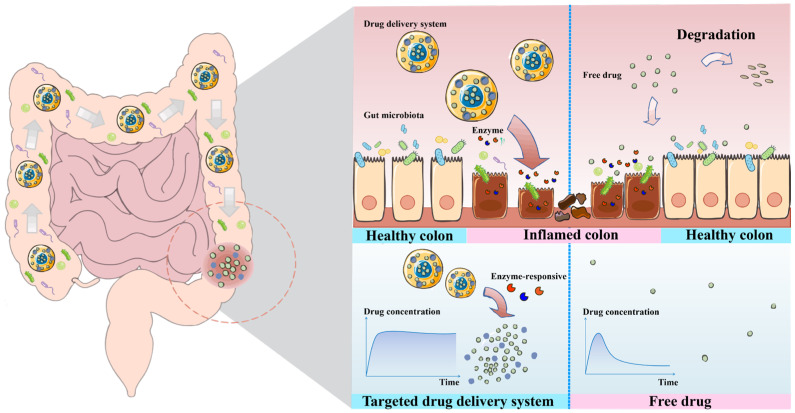
Existing IBD drug delivery systems tailored to colonic environment characteristics: optimize drug release directly at the inflamed sites within the colon. In the schematic, the thick gray arrows within the gastrointestinal tract indicate the transit pathway of the orally administered drug delivery system. The large curved red arrows highlight the enzyme-triggered degradation and activation of the polymeric carrier specifically at the inflamed colonic site, while the curved pink arrows indicate the two divergent fates of the free drug.

A growing body of evidence indicates that the intestinal microenvironment in IBD undergoes profound biochemical alterations, particularly in its enzymatic landscape, which closely correlate with disease activity [12]. In addition, the gut microbiota contributes to a highly heterogeneous intestinal metabolic milieu, resulting in variations in luminal enzyme expression among individuals [1]. In this context, enzyme-responsive polymeric drug delivery systems (DDSs) have therefore been developed as an intelligent therapeutic strategy capable of recognizing specific biochemical signals at inflammatory sites and subsequently triggering drug release [13]. Both natural polymers such as polysaccharides and proteins and synthetic polymers such as polyesters and polyacrylates can be engineered to respond to pathological stimuli present in the IBD microenvironment [14]. The core principle lies in harnessing certain specific enzymes that are significantly upregulated at the inflammatory sites of IBD as biological triggers. By conjugating or encapsulating drugs with polymer substrates that are selectively cleavable by these enzymes, the resulting DDSs remain stable in the normal gastrointestinal environment [15]. Drug release occurs only when the carriers reach diseased colonic tissues enriched with the corresponding enzymes, where enzymatic cleavage of the polymer structure triggers payload release [15]. This mechanism achieves on-demand and site-specific drug release (Figure 1). Compared with conventional free drug administration, this targeted strategy enables enhanced and prolonged accumulation of therapeutic agents specifically at inflamed intestinal lesions, thereby significantly improving the efficiency of IBD treatment [16].

Importantly, compared with other stimulus-responsive systems, enzyme-responsive vehicles are more closely linked to disease pathology and are less affected by physiological variability. Unlike pH-responsive systems, which are susceptible to variability across different regions of the gastrointestinal tract, and ROS-responsive systems, which may be activated under diverse inflammatory conditions, enzyme-responsive systems exploit disease-specific enzymatic signatures that are more closely associated with the pathological state of the inflamed colon. This enables more precise, stable, and predictable site-specific drug release.

In recent years, a growing body of research has explored diverse polymer architectures for enzyme-responsive IBD therapy, ranging from polysaccharide-based hydrogels and nanoparticles to synthetic polymer prodrugs and hybrid polymeric nanocarriers. Several recent reviews have summarized stimuli-responsive and colon-targeted drug delivery systems for inflammatory bowel disease [17,18,19]. For example, Long et al. provided a broad overview of nanoplatforms responsive to pH, enzymes, and reactive oxygen species [17], while other studies have focused on polymer prodrugs or polysaccharide-based carriers for colon-targeted delivery [18,19]. However, in these reports, enzyme-responsive systems are typically discussed as part of a broader stimuli-responsive framework, resulting in fragmented coverage and limited mechanistic depth. In particular, the critical relationship between polymer chemistry (e.g., backbone structure, functional groups, and crosslinking modes) and enzyme-specific degradation behavior has not been systematically analyzed. Moreover, the emerging concept of “drug–excipient synergy,” in which polymer degradation products actively contribute to therapeutic outcomes, has not been comprehensively addressed. To fill this gap, the present review provides a polymer-centric and mechanism-oriented perspective on enzyme-responsive drug delivery systems for IBD. We systematically correlate enzyme-triggered responses with polymer composition, molecular architecture, and degradation pathways, and further integrate these insights with therapeutic functionality. By doing so, this review establishes a unified framework that connects material design with biological performance, offering actionable design principles for the rational development of next-generation enzyme-responsive precision therapies for IBD.

## 2. Altered Enzyme Profiles in the Gastrointestinal Tract of IBD Patients Compared with Healthy Individuals

For enzyme-responsive polymeric DDSs, the fundamental distinction between the inflamed IBD gut and the healthy gut is not merely an isolated alteration in the activity of a single enzyme. Recent systems biology studies reveal the presence of a ‘functional dysbiosis’ in IBD, characterized by a disease-specific, cooperative remodeling of the functional repertoire of enzymes derived from the host, gut microbiota, and their interplay, culminating in a distinct pathogenic molecular milieu [20]. In the healthy gut, the enzymatic milieu is maintained in a state of homeostatic equilibrium, orchestrated to support digestion, barrier integrity, and immune tolerance; in IBD, this equilibrium is disrupted, and enzymatic activities are redirected toward sustaining inflammation, mediating tissue damage, and promoting aberrant repair [21]. Understanding the transition, characterized by the distinct enzymatic profiles unique to ulcerative colitis and Crohn’s disease as two disorders with divergent pathophysiology, establishes the logical foundation for the design of precisely targeted delivery systems [22].

**Figure 2:**
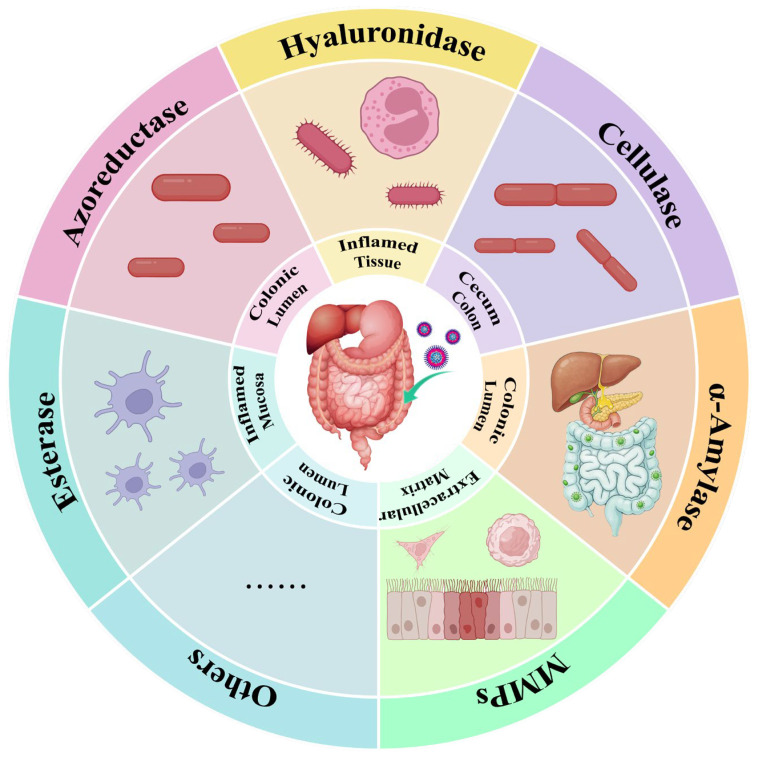
Classification and localization of enzymes associated with IBD.

Under the pathological conditions of IBD, specific anaerobic bacteria colonizing the colon are capable of secreting azoreductase, an enzyme that is virtually absent in the healthy small intestine. Although a large-scale metagenomic analysis found that the abundance of gut bacteria encoding this enzyme might be slightly higher in IBD patients compared to healthy individuals, the overall abundance remains low in both groups (Figure 2). Furthermore, there are differences in the functional diversity and activity of the enzyme. These factors are considered important contributors to the individualized clinical response observed with the traditional azo bond-based prodrug sulfasalazine (SAS) [23]. Beyond azoreductase, pectinase activity is also influenced by the composition of the colonic microbiota. The expression of pectinase is closely associated with the gut microbiome, suggesting that microbial differences between IBD patients and healthy individuals may lead to systematic alterations in enzymatic activity [24].

Regarding host-derived enzymes, the IBD-associated inflammatory microenvironment induces the dysregulated expression of a variety of enzymes. Studies have revealed that in the inflamed intestinal tissues of IBD patients, the activity of enzymes involved in extracellular matrix (ECM) remodeling is altered. For instance, elevated levels of key enzymes in the hyaluronan metabolism pathway, such as TSG-6, correlate with increased hyaluronan deposition and enhanced leukocyte infiltration in the tissue [25]. This provides a rationale for designing carriers that can be degraded by the highly active hyaluronidase present at inflammatory sites [26]. Simultaneously, the expression of esterases in activated macrophages at inflammatory sites may undergo alterations [27]. For instance, the expression of carboxylesterase Ces1d in macrophages is regulated by lipid signaling and participates in modulating their inflammatory status [28]. Studies on the tumor microenvironment have confirmed the overexpression of esterases, a property that has been leveraged to design responsive delivery systems [29]. A similar rationale may be applicable for inflammatory targeting in IBD [30]. Regarding cellulase [31] and α-amylase [32], existing literature has primarily focused on designing delivery systems based on their physiological characteristic of having higher basal activity in the healthy colon compared to the upper gastrointestinal tract. In contrast, precise comparative studies on their specific upregulation under IBD conditions remain to be further explored. Likewise, certain specific enzymatic activities share a similar physiological characteristic: they exhibit higher basal activity in the healthy colon compared to the upper gastrointestinal tract. Taking inulinase as an example, inulin possesses significant potential as an innovative colon-targeting carrier due to its unique metabolic process in the human body. It resists digestion and absorption in the stomach and small intestine, undergoing fermentation only in the colon via the action of inulinase—an enzyme exclusive to the colon [33,34]. Although comprehensive quantitative profiling of the IBD enzymatic microenvironment remains limited, several clinical studies provide representative data. Fecal MMP-9 levels are markedly elevated in active Crohn’s disease (≥0.34 ng/mL) and ulcerative colitis (≥0.36 ng/mL), with strong correlations with disease activity (AUC = 0.998 and 0.991, respectively, *p* < 0.001) [35]. Serum arylesterase activity is significantly reduced in IBD patients compared with healthy controls (963.9 ± 232.2 U/L vs. 1252.9 ± 275 U/L, *p* < 0.001) [36], indicating systemic alterations in esterase-associated pathways. Notably, for several key enzymes emphasized in enzyme-responsive delivery design, particularly microbiota-derived enzymes such as azoreductase, cellulase, and α-amylase, as well as hyaluronidase, quantitative comparisons between IBD and healthy cohorts are largely unavailable, with existing studies primarily reporting qualitative or semi-quantitative changes. This gap underscores a critical need for standardized and quantitative characterization of enzyme expression and activity to better inform the rational design of enzyme-responsive drug delivery systems.

A critical distinction exists between microbiota-derived and host-derived enzymes in their relative contributions to colonic drug release. Microbiota-derived enzymes serve as the primary drivers of colon-specific activation under physiological conditions. For instance, sulfasalazine shows negligible drug release in germ-free animals, demonstrating that bacterial azoreductase accounts for nearly 100% of its activation in healthy states [37]. In contrast, the contribution of host-derived enzymes is strongly dependent on inflammatory status. Under pathological conditions, the expression of enzymes is markedly upregulated in inflamed tissues, leading to a substantial increase in their role in drug activation. For example, in colitic rats, mucosal β-glucuronidase contributes up to 30–40% of dexamethasone prodrug hydrolysis, compared with less than 10% in healthy controls, where bacterial enzymes dominate [37]. These findings indicate that microbiota-derived enzymes provide baseline colon specificity, whereas host-derived enzymes act as inflammation-amplified co-triggers that enhance drug release specifically at diseased sites. This complementary and condition-dependent interplay represents an important design principle and has been increasingly leveraged in advanced delivery systems, including enzyme-triggered prebiotic nanoplatforms [38].

## 3. Enzyme-Responsive Polymeric DDS in IBD Therapy

Enzyme-responsive DDSs leverage the significant differences in enzyme distribution and activity across various segments of the gastrointestinal tract and under pathological conditions, offering an efficient solution to overcome physiological barriers and targeting challenges in the treatment of IBD. The core of this strategy lies in designing drug carriers containing enzyme-sensitive bonds or substrates that remain stable in the upper gastrointestinal environment yet are specifically recognized and degraded only upon reaching the colon by particular enzyme systems. These triggering enzymes are primarily derived from two sources: exogenous enzymes secreted by specific anaerobic flora colonizing the colon region (such as azo reductases and polysaccharide-degrading enzymes) and endogenous enzymes overexpressed by immune cells and damaged tissues in the inflammatory microenvironment (such as esterases and matrix metalloproteinases) (Table 1). Through such a precise “biological gating” mechanism, enzyme-responsive systems not only achieve site-specific burst release and concentration enrichment of drugs at the lesion sites, but also enable synergistic “drug excipient” therapeutic effects via the bioactivity of carrier degradation products. This section will elaborate in detail on the construction strategies and recent advances in various responsive systems, categorized according to the types of enzymes involved.

**Figure 3:**
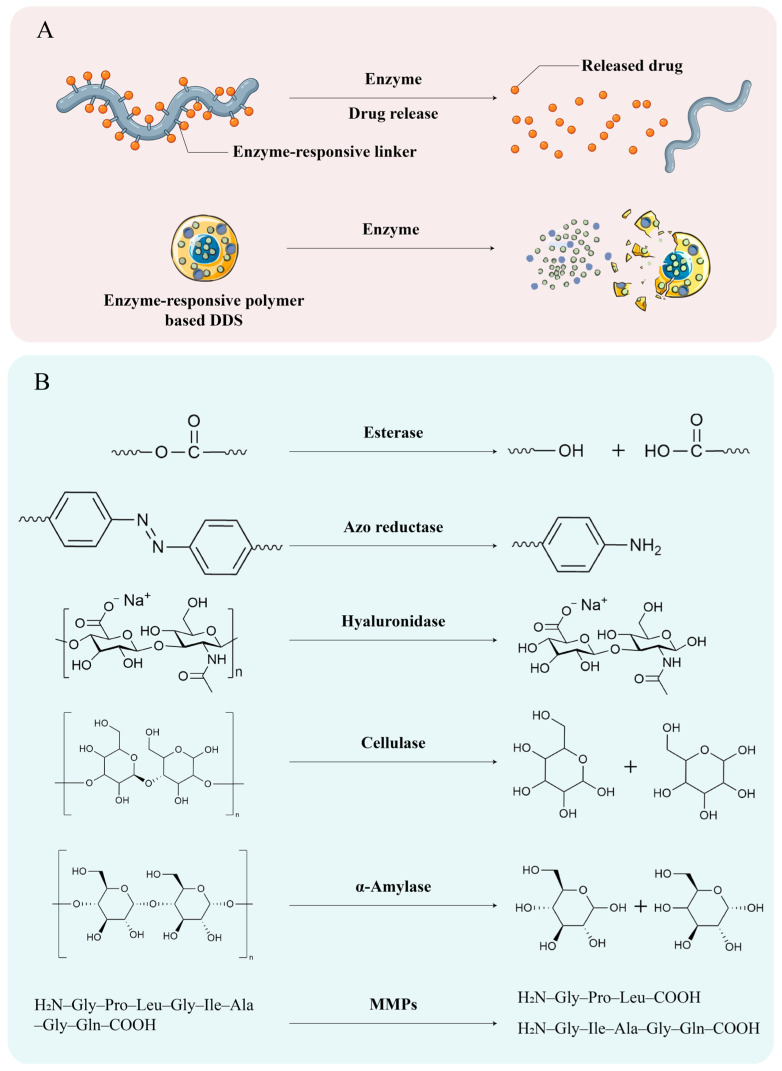
Chemical design principles of enzyme-responsive polymeric drug delivery systems for IBD. (**A**) Two fundamental release mechanisms: (i) covalent drug–polymer conjugates incorporating enzyme-cleavable linkers that enable direct, site-specific drug liberation upon enzymatic activation and (ii) enzyme-degradable polymer matrices that undergo structural disintegration to release physically encapsulated payloads. (**B**) Representative enzyme-sensitive chemical motifs and corresponding catalytic transformations, including ester hydrolysis (esterases), azo bond reduction to aromatic amines (azoreductases), glycosidic bond cleavage in hyaluronic acid (hyaluronidase), cellulose degradation (cellulase), and starch/cyclodextrin hydrolysis (α-amylase). These transformations illustrate the molecular basis of enzyme-triggered structural destabilization and controlled drug release.

As illustrated in Figure 3, enzyme-responsive polymeric drug delivery systems operate through two principal mechanisms. In the first approach (Figure 3A, left pathway), therapeutic agents are covalently attached to the polymer backbone via an enzyme-labile linker. Following exposure to disease-associated enzymes overexpressed in the inflamed colon, the linker is selectively cleaved, releasing the free drug. In the second approach (Figure 3A, right pathway), drugs are physically encapsulated within a polymer matrix that is intrinsically susceptible to enzymatic degradation. Here, the polymer itself acts as the responsive element; enzymatic hydrolysis of the polymer backbone destabilizes the carrier and triggers drug release. The schematic representation highlights a polymer chain with a pendant drug molecule connected through a cleavable linkage—upon enzyme action, the bond is broken and the drug is liberated. Figure 3B summarizes the key enzyme–substrate pairs exploited in current IBD-targeted systems. These include esterase (cleaving ester bonds), azoreductase (reducing azo bonds to amines), hyaluronidase (degrading hyaluronic acid), cellulase (hydrolyzing cellulose derivatives), and α-amylase (degrading starch-based or cyclodextrin polymers). The chemical transformations shown (e.g., generation of hydroxyl or amino groups) underscore the molecular basis for enzyme-triggered drug release. Collectively, this figure provides a visual framework for understanding how polymer chemistry and enzyme specificity are harnessed to achieve spatiotemporally controlled drug delivery in IBD therapy.

From a mechanistic perspective, a critical design feature of these enzyme-responsive platforms is the dynamic transition from diffusion-controlled to enzyme-triggered release. During transit through the upper gastrointestinal tract, the dense crosslinking or steric bulk of the stable polymer network tightly restricts the diffusion coefficient of the encapsulated drug, effectively minimizing premature leakage (diffusion-controlled phase). Upon reaching the colon, specific enzyme substrate interactions cleave the structural backbone or crosslinkers. This targeted degradation leads to a rapid relaxation, swelling, or complete collapse of the polymeric matrix, fundamentally altering the mass transport kinetics and initiating a massive, site-specific burst release of the therapeutic cargo (enzyme-triggered phase). Understanding this transition is essential for optimizing degradation kinetics and therapeutic efficacy.

### 3.1. Esterase-Responsive DDS

The infiltration of immune cells (particularly macrophages and monocytes) in the colon region leads to the secretion of substantial amounts of esterase, resulting in significantly higher esterase activity in inflammatory tissues compared to healthy tissues. Leveraging this pathological discrepancy, DDSs containing ester bonds can be designed to achieve the specific, “on-demand” release of drugs at the lesion site. This strategy not only prevents premature drug leakage in the stomach and small intestine but also synchronizes carrier degradation with drug release through enzymatic cleavage cascades, thereby greatly enhancing the precision and safety of treatment [57].

A core strategy involves covalently linking drug molecules or bioactive lipids via ester bonds to polymer backbones to create polymer nanocarriers that are stable under physiological conditions but unstable in the inflammatory enzymatic environment. For instance, polymer prodrugs can be designed by conjugating anti-inflammatory natural products (such as quercetin) with polymeric lipid chains (e.g., modified medium-chain triglycerides) via ester bonds, enabling their assembly into esterase-sensitive nanocarriers. Crucially, this prodrug architecture provides a distinct pharmaceutical advantage in drug loading efficiency. As the therapeutic molecule constitutes part of the carrier backbone, drug loading is stoichiometric and precisely controlled, eliminating issues of low encapsulation efficiency and premature leakage associated with conventional nanocarriers. The structural parameters of such core–shell nanocarriers critically dictate their performance. For example, researchers designed a core–shell system using Qu-SS-Gcc lipid nanoparticles as the core and chitosan as the shell. By optimizing the design parameters, a shell-to-core mass ratio of 1:1 yielded uniform nanoparticles with a hydrodynamic diameter of ~162 nm and high encapsulation efficiency (>93%). The chitosan shell provides stability during gastrointestinal transit, while esterase-triggered degradation of the lipid core significantly enhances drug release (from 24% to 49% within 48 h) [40]. This “structural collapse–drug release” mechanism effectively addresses the issue of slow drug release from traditional lipid carriers at the lesion site.

**Figure 4:**
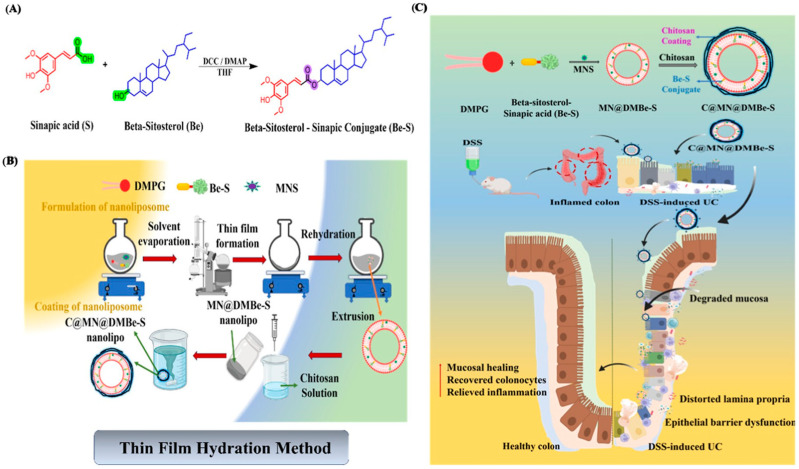
Enzyme-responsive chitosan armored beta sitosterol sinapic acid conjugate nanoliposomes as dual therapy for DSS-induced colitis. (**A**) Synthetic scheme for the synthesis of the Be–S conjugate. (**B**) Formulation and preparation of nanoliposomes by the thin-film hydration method. (**C**) Mechanistic illustration of oral delivery: chitosan coating ensures gastric stability and protects the payload during transit. In the colon, enzymatic degradation of chitosan exposes negatively charged nanoliposomes, enhancing electrostatic interaction with inflamed mucosal proteins. This promotes localized retention and enables sustained, enzyme-triggered drug release, leading to effective anti-inflammatory activity and mucosal repair. Re-adapted with permission from Reference [30]. Copyright American Chemical Society 2025.

To further enhance therapeutic efficacy, esterase-responsive strategies have evolved from simple carrier degradation to integrated “prodrug-carrier” co-delivery systems. This involves coupling two pharmacologically active molecules via esterification to form a prodrug, which not only improves the physicochemical properties of the drugs (such as solubility or stability) but also enables the in situ regeneration of bioactive molecules upon esterase action. For instance, a conjugate formed by esterifying β-sitosterol with sinapic acid can be integrated into polymeric membrane materials or serve as a component of a polymeric carrier system. This design not only provides structural stability to the polymeric carrier during gastrointestinal transit, but, more crucially, upon guidance by a chitosan coating to the colon inflammatory site, the esterase in the environment specifically cleaves the ester bond between β-sitosterol and sinapic acid. This process releases free β-sitosterol and sinapic acid on demand, exerting the antioxidant effect of sinapic acid and indirectly promoting the production of short-chain fatty acids (SCFAs) by improving the intestinal microenvironment. Concurrently, the enzymatic loosening of the polymeric membrane structure promotes the sustained release of internally encapsulated NLRP3 inflammasome inhibitors (e.g., MNS) (Figure 4) [30]. This strategy not only overcomes the challenge of low bioavailability of hydrophobic drugs but also achieves the spatiotemporally synchronized delivery of antioxidants, anti-inflammatory agents, and microbiota modulators via the enzyme-responsive mechanism.

Building on the aforementioned foundations, the latest research focuses on developing multifunctional gel systems aimed at resolving the conflict between undesirable sensory properties of drugs (e.g., unpleasant odor) and precise targeting through esterase-responsive mechanisms, and achieving deep synergy between metabolic reprogramming and immune regulation. By synthesizing prodrugs via ester bonds from short-chain fatty acids with strong odors and irritation (e.g., butyric acid) and flavonoids (e.g., apigenin), and encapsulating them within esterase-responsive hydrogels (e.g., ascorbyl palmitate/alginate), oral microbead systems with a “dual esterase lock” can be constructed. Crucially, the mechanical resilience and in vivo stability of macroscopic hydrogels during gastrointestinal transit are decisive for their therapeutic performance. In this system, the alginate-based network is reinforced by dense Zn^2+^-mediated ionic crosslinking, forming a mechanically robust framework that limits water infiltration and polymer relaxation. This architecture enables the microbeads to maintain complete structural integrity in highly acidic gastric fluid (pH 2.0) for up to 48 h and to withstand the combined chemical and mechanical stresses encountered in the upper gastrointestinal tract in vivo [39]. Such stability ensures efficient delivery to the colonic target site prior to enzyme-triggered degradation.

Subsequently, esterase further hydrolyzes the ester bond within the prodrug molecule, releasing free butyric acid and apigenin. This cascade enzymatic cleavage mechanism ensures the precise accumulation of drugs within inflammatory cells, which then synergistically inhibits the CK2-NF-κB signaling pathway and activates PPAR-γ-mediated metabolic reprogramming, fundamentally restoring intestinal barrier function and modulating microecological balance [39]. This multi-level, multi-target esterase-responsive design represents the latest development direction for precise oral drug DDSs in IBD.

### 3.2. Azoreductase-Responsive DDS

Unlike esterase, which is widely expressed throughout the gastrointestinal tract and other tissues, the distribution of azoreductase is highly colon-specific [58]. This specificity primarily stems from the metabolic production of this enzyme system by the high-abundance anaerobic microbiota (such as Bacteroides and Clostridium genera) colonizing the distal colon. From a chemical mechanism perspective, azoreductase can act as an electron donor, mediating the reductive cleavage of aromatic azo bonds (–N=N–) to generate corresponding aromatic amines. This unique enzymatic catalytic process establishes the azo bond as the core “chemical switch” in colon-targeted delivery systems [37]. However, the release kinetics of such prodrugs remain a key limitation. Steric hindrance imposed by the polymer backbone can significantly restrict enzyme accessibility to cleavable bonds, often resulting in slow or incomplete drug activation. This challenge necessitates rational molecular design strategies, such as spacer insertion and linker optimization, to achieve efficient and timely drug release at inflamed sites.

To circumvent the risk of non-specific degradation of single azo bonds in the gastric acidic environment or during small intestinal transit, modern delivery system design tends to incorporate “steric shielding” or “dual-gating” mechanisms. A typical strategy involves constructing polymeric core–shell-structured nanocarriers, using pH-sensitive polymers (such as Eudragit S100) as the outer layer to encapsulate the azo-bond-containing polymeric polyurethane core. This design leverages the insolubility of the pH-sensitive layer in the stomach and small intestine to provide a physical barrier for the internal polymer structure. Upon reaching the ileocecal region and colon (pH > 7.0), the outer polymer layer dissolves, exposing the core. Subsequently, the azo bonds are cleaved by bacterial enzymes, leading to the structural disassembly of the polymer nanoparticles and thereby achieving site-specific burst release of the drug. This mechanism, through the tandem response of pH and enzyme, effectively addresses the challenge of premature drug release at the terminal ileum due to elevated pH [42]. In the design of carriers for poorly soluble drugs, utilizing azo bonds to control the crosslinking density of polymer networks is another key strategy. Researchers have synthesized azo group-containing dimethacrylate crosslinkers (DMAAB) to construct hydrogel networks. This covalent network architecture confers excellent gastrointestinal stability, as the dense crosslinked structure effectively suppresses drug diffusion during transit through the stomach and small intestine, thereby minimizing premature release prior to reaching the colonic target. These networks maintain a dense mesh structure in the physiological environment, restricting the diffusion of drug molecules.

Advancing beyond macroscopic hydrogels, the engineering of self-assembled polymeric nanomicelles represents a highly effective strategy for azoreductase-responsive delivery. A representative example involves colon-targeted, mucoadhesive micellar nanocarriers for curcumin delivery [43]. In this system, an amphiphilic block copolymer containing azo linkages (PEG-Azo-PLGA) is co-assembled with catechol-modified TPGS (Cat-TPGS). The catechol groups enhance mucoadhesion, prolonging retention at inflamed colonic sites. Upon exposure to colonic azoreductases, the azo bonds are cleaved, leading to micelle disassembly and site-specific drug release. This system significantly alleviates colitis by downregulating pro-inflammatory cytokines (MPO, IL-6, IL-1β, TNF-α) and modulating the TLR4/MyD88/NF-κB signaling pathway while also contributing to microbiota homeostasis.

However, upon contact with colonic flora, the enzymatic cleavage of the azo crosslinking points triggers a dramatic increase in the swelling rate or even degradation of the polymer network. This structural relaxation effect rapidly releases the entrapped hydrophobic drugs (e.g., mesalazine) [41]. To further enhance therapeutic efficiency, a negatively charged alginate skeleton can be employed to target and adsorb onto positively charged inflamed mucosal surfaces via electrostatic interactions while covalently linking the drug–prodrugs via azo bonds. This design not only prolongs retention time through mucosal adhesion but also converts inactive prodrugs into active drugs in situ via enzymatic reactions, achieving high local drug concentration maintenance at the lesion site [59].

A representative example is the development of azo-linked hydrogel microparticles for sustained delivery of 5-aminosalicylic acid (5-ASA) [59]. In this system, the therapeutic agent is covalently conjugated to a hydrophilic polymer backbone via azo linkages, forming a crosslinked hydrogel network. This architecture serves a dual function: the dense polymer network protects the azo bonds from premature degradation in the upper gastrointestinal tract, while the swollen hydrogel matrix provides mucoadhesive properties that prolong retention at inflamed colonic sites. Upon reaching the colon, azoreductases produced by the gut microbiota selectively cleave the azo bonds, leading to degradation of the polymer network and triggering sustained drug release. This enzyme-responsive mechanism ensures localized delivery and maintains high drug concentrations at the site of inflammation.

Despite the elegant design of azoreductase-triggered systems, their clinical translation is fundamentally constrained by pronounced inter-patient variability in gut microbiota composition. The reductive cleavage of azo bonds is strictly dependent on the presence and metabolic activity of specific obligate anaerobes (e.g., Bacteroides and Clostridium) [60]. However, IBD is typically associated with severe microbial dysbiosis, often leading to a reduced abundance and altered functionality of these azoreductase-producing species [61]. As a result, inter- and intra-patient variability in colonic enzymatic activity can give rise to highly inconsistent pharmacokinetic profiles, potentially causing premature drug release or, conversely, incomplete drug activation. To mitigate this limitation, next-generation azoreductase-responsive systems should incorporate strategies aimed at improving robustness and personalization. These include the co-delivery of prebiotic components to selectively enrich azoreductase-producing microbial populations, as well as the integration of companion diagnostics (e.g., fecal microbiome profiling) to enable patient stratification and precision therapy. In contrast, host-derived enzyme-responsive systems leverage inflammation-associated enzyme overexpression within diseased tissues, providing a more stable and pathology-driven trigger mechanism that is less susceptible to microbiota variability (e.g., systems targeting hyaluronidase or matrix metalloproteinase).

### 3.3. Hyaluronidase-Responsive DDS

The specificity of HA-based carriers primarily stems from their specific binding to the CD44 receptor. Under colonic inflammatory conditions, the expression of CD44 receptors on the surfaces of macrophages and epithelial cells is significantly upregulated, providing a molecular basis for the active targeting of HA nanocarriers [62]. Studies have shown that self-assembled HA nanoparticles constructed via amphiphilic modification can be efficiently internalized by CD44-positive cells (such as inflammatory Caco-2 cells and activated macrophages) predominantly aggregated at inflammation sites through receptor-mediated endocytosis. Their uptake efficiency is significantly higher than that by non-inflammatory cells, thereby achieving precise drug enrichment at the lesion site [44]. Further mechanistic studies reveal that this targeting effect is not limited to cellular entry. HA modification can also competitively block endogenous inflammatory signaling pathways, such as inhibiting the activation of the TLR4/MyD88/NF-κB signaling axis, through its interaction with CD44, thereby exerting synergistic anti-inflammatory effects while delivering drugs [45].

However, constructing hyaluronidase (HAase)-responsive systems faces the challenge of balancing stability in the upper gastrointestinal tract with enzymatic responsiveness in the colon. After oral administration, HA carriers must resist non-specific destruction in the gastric acid and small intestinal environments to reach the colon region rich in both bacterial and host-derived HAase. The selection of molecular weight plays a key role in this balance. High-molecular-weight HA (HMW-HA) demonstrates superior stability in simulated gastric and intestinal fluids compared to low-molecular-weight HA, while it can be rapidly degraded by HAase in simulated colonic fluid, triggering burst drug release. For instance, core–shell-structured nanoprobes coated with HMW-HA can not only remain intact in the upper GI tract but also undergo structural disintegration in the colon in response to HAase, releasing curcumin and cerium oxide nanozymes to achieve reactive oxygen species (ROS) scavenging at inflammatory sites and real-time CT imaging monitoring [26].

To further enhance the stability of HA carriers in the complex gastrointestinal environment and achieve more precise enzyme-responsive release, strategies involving multiple modifications and multi-layer coatings are widely employed. For example, introducing calcium pectinate (CP) as an outer protective shell can effectively prevent premature degradation of HA before it reaches the colon. Upon arrival in the colon, the colonic microbiota first degrades the CP layer, exposing the inner HA layer. Subsequently, the HA layer is further degraded by the high concentration of HAase at the lesion site, releasing the drug. Simultaneously, the exposed lactoferrin ligands enable dual targeting of intestinal epithelial cells [35]. Furthermore, nanomedicines formed by chemically conjugating hydrophobic drugs (e.g., bilirubin) directly onto the HA backbone leverage the hydrophobic stacking effect of the bilirubin core. This modification not only endows the carrier with enhanced antioxidant and anti-inflammatory properties but also partially retards its degradation by HAase, thereby prolonging drug residence time on the inflamed mucosa. Importantly, this system provides a compelling example of the concept of bioactive degradation products. Robust in vivo evidence demonstrates that the biological processing of these HA conjugates actively remodels the gut microbiome architecture, most notably by restoring the relative abundance of the beneficial mucin-degrading bacterium Akkermansia muciniphila from near depletion in untreated colitis to approximately 20%. This carrier-mediated microbiome modulation directly correlates with accelerated mucosal healing and significant suppression of pro-inflammatory cytokines, including TNF-α and IL-6 [46].

Notably, in HAase-responsive systems, HA not only acts as an inert carrier or targeting ligand but its own degradation products and biological activities also positively impact IBD treatment. Although low-molecular-weight HA fragments are considered pro-inflammatory in certain contexts, exogenous administration of HA and its enzymatic degradation process have been shown, in specific models of intestinal infection and injury, to resist pathogen invasion and promote mucosal healing by enhancing intestinal barrier function, promoting antimicrobial peptide secretion, and modulating immune cell differentiation [63,64]. Moreover, after degradation by HAase, HA carriers can penetrate more deeply into the submucosa of inflamed tissues, restoring intestinal epithelial integrity by regulating the expression of tight junction proteins (such as ZO-1 and Occludin) [65].

In summary, hyaluronidase-responsive DDSs, by integrating CD4+ active targeting, enzyme-triggered drug release, and the inherent mucosal repair function of HA, provide a multi-dimensional solution for IBD therapy. Future research should further focus on achieving intelligent responsiveness to variations in HAase concentration across different stages of intestinal disease through precise control of the chemical modification degree and molecular weight of HA.

### 3.4. Cellulase-Responsive DDS

The construction of cellulase-responsive delivery systems is fundamentally based on the significant enzymatic profile difference between the host and the intestinal microbiota. Mammals themselves lack the enzyme systems capable of hydrolyzing β-1,4-glycosidic bonds [66]. However, early microbiological studies confirmed that the colon harbors a high density of cellulolytic bacteria. For instance, in rat models, the density of cellulolytic bacteria in the cecum can reach 0.5 × 10^8^–12.2 × 10^8^/g, predominantly Bacteroides succinogenes, which is far higher than that in the terminal ileum (approximately 10^3^/g) [67]. This specific spatial gradient in enzymatic activity makes cellulose and its derivatives ideal “gatekeeping” materials for colon targeting.

Leveraging this physiological characteristic, researchers initially focused on constructing “enzyme-responsive switches” through surface engineering to address the premature release of oral drugs in the upper gastrointestinal tract. Using the layer-by-layer (LbL) self-assembly technique, a dense polyelectrolyte complex layer can be formed on the surface of nanocarriers by alternating negatively charged NaCS and positively charged chitosan quaternary ammonium salt, thereby providing a physical shield for the drug core. This core–shell structure remains intact in the non-enzymatic environments of the stomach and small intestine, effectively blocking drug diffusion. Upon entry into the colon, however, the highly active cellulase specifically degrades the glycosidic bonds within the NaCS backbone, leading to shell disintegration and triggering the site-specific burst release of drugs (e.g., budesonide) [31]. This mechanism not only enhances the bioavailability of the drug at the lesion site but also, due to the nanoscale size and surface charge properties of the carrier, further improves its retention capacity in inflamed tissues.

Beyond serving as a surface coating, cellulose nanomaterials are frequently employed as reinforcing agents for microparticle skeletons to modulate the swelling and degradation kinetics of carriers, owing to their excellent mechanical properties and network-forming capabilities. For example, introducing CNF into a retrograded starch/pectin microsphere system allows the construction of a tight supramolecular network within the polymer matrix, leveraging the high aspect ratio and abundant hydroxyl groups of CNF. This dense structure, on the one hand, retards water molecule penetration, thereby delaying microsphere swelling and drug leakage in simulated gastric fluid. On the other hand, the exposed hydroxyl groups on CNF significantly enhance the carrier’s adhesion to the colonic mucosa. When the CNF content is 50%, the adhesion force reaches a maximum of 3.40 N, showing an increasing trend with higher CNF proportions, which extends the drug’s effective window [48]. Furthermore, utilizing its unique three-dimensional nanofiber network, bacterial cellulose (BC) can be combined with materials like sodium alginate to form semi-interpenetrating network hydrogels. This not only improves the structural stability of the carrier but also provides a structural foundation for building multiple-stimuli-responsive (e.g., pH/enzyme synergistic) drug delivery platforms [47].

The core advantage of cellulase-responsive systems in the treatment of IBD lies in their intelligent transformation between “structural protection” and “enzymatic collapse”: prior to reaching the target site, cellulose materials act as physical barriers or skeleton reinforcers to ensure carrier stability; upon reaching the colon, they are rapidly converted into metabolic substrates for bacteria, thereby achieving precise drug release. Future optimization directions primarily involve the precise regulation of the degree of substitution and composite ratios of cellulose derivatives to further match the enzymatic activity levels of the intestinal microbiota under different disease conditions.

### 3.5. α-Amylase-Responsive DDS

α-Amylase is an endoenzyme capable of specifically hydrolyzing internal α-1,4-glycosidic bonds in polysaccharide chains, widely present in human digestive fluids and intestinal microbial metabolites. In the therapeutic strategies for IBD, utilizing α-amylase as a trigger to construct colon-targeting DDSs is primarily based on the differential enzymatic degradation of specific polysaccharide carriers at distinct physiological sites and the specific changes in enzyme levels under IBD pathological conditions [68].

Starch, as a natural polysaccharide with good biocompatibility and biodegradability, serves as a foundational material for constructing such systems. However, natural starch is highly susceptible to rapid degradation by pancreatic amylase in the stomach and upper small intestine, leading to premature drug release. To address this issue, RS has been introduced as a colon-targeting carrier. Through physical modification (e.g., retrogradation treatment forming RS3) or chemical modification, RS can effectively resist acidolysis and enzymolysis in the upper gastrointestinal tract until it reaches the colon. In the colon, abundant intestinal microbiota secretes bacterial amylases (including α-amylase), which can specifically degrade the RS coating or matrix, thereby achieving site-specific drug release. For instance, in a starch-based core–shell microsphere system, shell formulation parameters critically determine degradation kinetics. A double-coating strategy achieving a 50% (*w*/*w*) shell thickness, combined with 10% high-crystallinity native starch (RS2), effectively restricted premature drug leakage in the upper gastrointestinal tract (~40%) while enabling targeted release in the colonic enzymatic environment [51].

**Figure 5:**
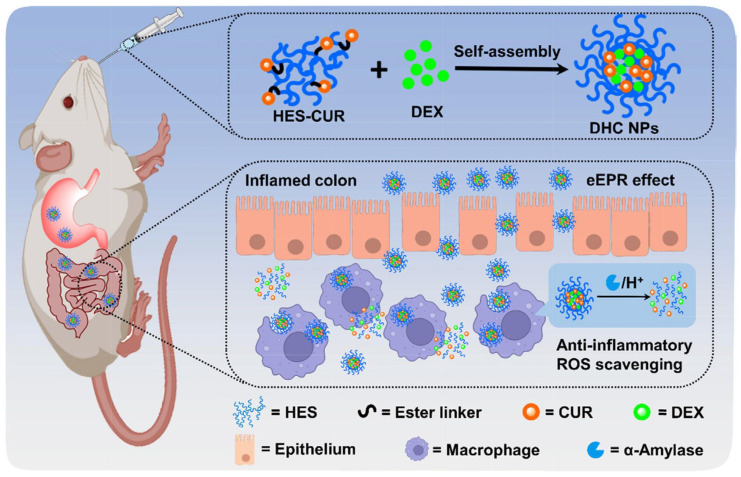
Schematic illustration of the fabrication and oral-targeted delivery mechanisms of DHC nanoparticles (NPs). (**Top**) Construction of DHC NPs through self-assembly of hydroxyethyl starch (HES)–curcumin (CUR) conjugates and dexamethasone (DEX). (**Bottom**) Sequential in vivo mechanism: gastrointestinal transit and passive targeting to inflamed tissue via the eEPR effect; cellular uptake by activated macrophages; and multi-triggered degradation within the inflammatory microenvironment. Enzymatic (α-amylase), acidic, and esterase-mediated cleavage collectively induce nanoparticle destabilization and co-release of DEX and CUR, resulting in synergistic anti-inflammatory and antioxidant effects. Re-adapted with permission from Reference [49]. Copyright Elsevier 2022.

Beyond utilizing enzymes produced by the gut microbiota, the pathological characteristics of colonic tissue in IBD patients also provide a novel target for designing α-amylase-responsive systems. Research has found that during the pathogenesis of ulcerative colitis (UC), accompanying pancreatic complications can lead to abnormally elevated levels of α-amylase in the colonic lumen. Based on this pathological feature, HES, a semi-synthetic polysaccharide, has garnered attention due to its good water solubility and specific responsiveness to α-amylase. The α-1,4-glycosidic bonds in the HES backbone can be specifically cleaved by α-amylase, leading to carrier disintegration. By covalently conjugating hydrophobic drugs (e.g., curcumin or dexamethasone) with HES (such as via ester bond formation in CUR-HES conjugates) or encapsulating them within HES-based carriers, self-assembled nanoparticles or microspheres can be formed. This not only significantly improves the solubility and stability of the drugs but also allows for triggered drug release by the highly expressed α-amylase at the inflammatory site. This strategy has been validated in a DSS-induced mouse colitis model: upon reaching the inflamed colon, the drug delivery system degrades under the action of high concentrations of α-amylase, achieving precise drug release (Figure 5). Simultaneously, the inherent properties of the carrier material itself (e.g., the antioxidant capacity of the HES-CUR conjugate) provide a synergistic therapeutic effect [49,52].

Furthermore, cyclodextrin (CD), a cyclic oligosaccharide possessing a hydrophobic internal cavity and a hydrophilic outer surface, can be integrated into polymeric systems as a “gating” component for constructing α-amylase-responsive carriers. Cyclodextrins can not only encapsulate hydrophobic drugs via host–guest recognition to enhance their bioavailability, but their α-1,4-glycosidic bond structure is also sensitive to α-amylase [50]. In a simulated colonic environment, α-amylase can disrupt the cyclic structure of cyclodextrin, leading to the dissociation of the inclusion complex and subsequent drug release. To further improve targeting precision, researchers often combine cyclodextrins with other polymeric responsive moieties (e.g., ROS-responsive groups integrated into a polymer backbone) to construct multi-responsive polymeric nanoplatforms. For example, using phenylboronic acid-modified cyclodextrin as a component within a polymeric carrier system can not only respond to excess ROS at inflammatory sites but also accelerate drug release under the action of α-amylase, alleviating colitis symptoms through multiple mechanisms such as modulating gut microbiota and repairing the intestinal barrier [32,50].

### 3.6. Matrix Metalloproteinase-Responsive DDS

In contrast to the aforementioned enzymes secreted by the gut microbiota, such as azoreductase and polysaccharide-degrading enzymes, matrix metalloproteinases (MMPs) constitute a family of zinc-dependent endopeptidases secreted by host cells, with their primary function being the degradation of the ECM. In the healthy gastrointestinal tract, MMP activity is tightly regulated by tissue inhibitors of metalloproteinases (TIMPs), maintaining a dynamic equilibrium at a low level. However, under the pathological conditions of IBD, this balance is severely disrupted, leading to the specific high expression of MMPs at inflammatory sites. This pronounced spatiotemporal variation in enzyme activity provides a precise “biological gating” mechanism for constructing targeted delivery systems designed for the inflammatory tissue microenvironment.

A deeper investigation into its pathological basis reveals that the sources of MMPs in the intestines of IBD patients are diverse and are tightly regulated by immune signaling. In addition to traditional immune cells, intestinal fibroblasts have been confirmed as significant sources of MMPs. Studies have shown that in patients with Crohn’s disease and ulcerative colitis, the T cell-derived cytokine interleukin-21 (IL-21) is significantly upregulated. By binding to the IL-21 receptor on the surface of fibroblasts, it specifically induces, at a posttranscriptional level, the secretion of MMP-1, MMP-2, MMP-3, and MMP-9. This process can synergize with TNF-α, leading to exacerbated tissue destruction [69]. Furthermore, intestinal epithelial cells, serving as the first line of the mucosal barrier, are also involved in this process. Inflammatory cytokines (e.g., TNF-α) can stimulate epithelial cells to overexpress MMP-1, MMP-3, MMP-9, etc., resulting in impaired epithelial barrier function and ulcer formation [70]. Beyond directly degrading the ECM, MMP-7 (Matrilysin) has been found to specifically degrade the tight junction protein Claudin-7. This process directly compromises the barrier function of the intestinal epithelium, leading to the permeation of luminal antigens and further aggravating the inflammatory response, creating a vicious cycle [71].

Regarding the specific enzymatic profile, different MMP subtypes are closely related to disease activity. Analysis of clinical samples indicates that the levels of MMP-3 in serum and tissue show a significant positive correlation with disease activity indices in patients with ankylosing spondylitis complicated by IBD, suggesting MMP-3 can serve as a sensitive biomarker for assessing inflammatory severity [72]. In ulcerative colitis, the overexpression of MMP-9 is particularly prominent. Research has found that serum levels of Cyclophilin A (CyPA) are significantly elevated in ulcerative colitis patients and positively correlate with the high expression of MMP-9 in tissues. CyPA may interact with its receptor CD147, activating the ERK1/2 signaling pathway, thereby disrupting the TIMP-1/MMP-9 balance and promoting the release of MMP-9 and inflammatory infiltration [73].

Based on the pathological features described above, smart hydrogel systems crosslinked with MMP-sensitive peptides have emerged. For example, researchers developed an “inflammation-targeting hydrogel (IT-hydrogel)” formed by the self-assembly of ascorbyl palmitate. This hydrogel carries a negative surface charge, enabling it to specifically adhere via electrostatic interactions to the positively charged inflamed mucosal surface, where it is subsequently degraded by the highly expressed MMPs at the inflammatory site, releasing the encapsulated anti-inflammatory drug (e.g., dexamethasone). This dual mechanism of “adhesion enzymolysis” significantly reduces systemic drug exposure and enhances local therapeutic efficacy [53]. To withstand continuous mechanical shear from intestinal peristalsis while enabling precise on-demand release, an MMP-9-responsive hydrogel based on gelatin and multi-arm PEG has been developed. The mechanical behavior and in vivo stability of this system are governed by its dynamic network architecture. By tuning the PEG arm number (2-, 4-, or 8-arm) and crosslinking density, the storage modulus (G′) can be adjusted to approximately 1000 Pa, closely matching that of native colonic tissue. Moreover, dynamic hydrazone crosslinks impart rapid shear-thinning and self-healing properties, allowing instantaneous recovery following high strain (100%), thereby conferring strong resistance to mechanical disruption. This optimized mechanical profile enables sustained colonic retention (12–36 h) while simultaneously regulating hydrogel degradation and drug release kinetics. This tunable carrier not only releases 5-ASA in response to MMP-9 concentration but also, by acting as a “substrate trap” for MMP-9, helps alleviate inflammatory damage by sequestering excess MMP-9 [54].

In summary, the specific enrichment of MMPs in IBD lesions is not coincidental but a direct consequence of dysregulated host immune responses. Leveraging this pathological feature, particularly by designing smart responsive carriers targeting key enzymes such as MMP-9 and MMP-7, not only achieves site-specific drug release but also may exert synergistic therapeutic effects by modulating the enzyme activity itself.

### 3.7. Other Enzyme-Responsive DDS

Beyond esterases specifically expressed in inflammatory tissues, the abundant repertoire of microbial enzymes (such as glycosidases like inulinase and pectinase) in the colonic microenvironment offers another efficient pathway for achieving colon-targeted delivery. Unlike the upper gastrointestinal tract, which lacks enzymes capable of degrading complex polysaccharides, the anaerobic microbiota in the colon can selectively hydrolyze polymers such as inulin, pectin, and dextran. Dextran, for example, is a glucose-based polymer specifically degraded by colonic dextranases via cleavage of α-1,6-glycosidic linkages, making it a prototypical backbone for colon-targeted prodrugs and hydrogel systems. DDSs constructed by leveraging this characteristic not only protect drugs from gastric acid degradation but also utilize the “prebiotic” properties of polysaccharide degradation products to modulate the gut microbiome, realizing a “drug adjuvant synergistic” therapy.

Inulin, a classic prebiotic polysaccharide, is widely utilized in carrier construction due to its degradability by the colon-specific enzyme inulinase. To address the issue of strong hydrophilicity in natural inulin, which makes it difficult to encapsulate hydrophobic drugs, chemical modification serves as an effective strategy. For instance, grafting the hydrophobic dehydro-peptide (Boc-Phe-∆Phe-ƐAhx-OH) onto the inulin backbone via ester bonds can construct amphiphilic inulin–peptide conjugates. These conjugates can self-assemble in aqueous media to form nanostructures with a core–shell architecture, encapsulating hydrophobic drugs like ornidazole within the core. When these nanostructures reach the colon, the environmental inulinase specifically hydrolyzes the β-2,1-glycosidic bonds in the inulin backbone, leading to the disassembly of the micellar structure and rapid drug release, thereby achieving site-specific drug accumulation at the lesion site [56].

Beyond serving as a carrier backbone, inulin is often designed as a “gating” coating for polymeric nanocarriers to construct “switch”-type release systems. For example, modifying polymeric nanoparticles with carboxymethyl inulin can effectively block the pores, preventing premature leakage of drugs (e.g., budesonide) in the stomach and small intestine. Once the polymeric particles reach the colon, the polymeric inulin layer on the surface is degraded by microbiota-secreted inulinase, opening the pore “switch” and triggering drug release. More importantly, the degradation products of the inulin polymer layer, acting as prebiotics, can significantly promote the proliferation of beneficial bacteria like Bifidobacterium and Lactobacillus while inhibiting pathogenic bacteria. This dual action of modulating gut microbiota and releasing anti-inflammatory drugs is significantly superior to single-drug therapy [15].

Similar to inulin, polysaccharides such as pectin and Nutriose (a resistant dextrin) are also frequently used as preferred materials for constructing enzyme-responsive shells, and their degradation products exhibit unique immunomodulatory functions. Using the layer-by-layer (LbL) self-assembly technique, a drug-loaded PLGA core can be coated via electrostatic interactions between pectin and chitosan to form nanoparticles with dual pH and enzyme sensitivity. The pectin/chitosan shell of this system contracts in gastric acid to protect the encapsulated drug and undergoes selective degradation in the colon. Mechanistically, pectinases catalyze the hydrolysis of α-1,4-D-galacturonic acid linkages within the pectin backbone, triggering shell disassembly and drug release. Importantly, the resulting pectin-derived oligosaccharides exhibit intrinsic bioactivity, contributing to therapeutic outcomes. Experimental evidence demonstrates that these degradation products promote immune rebalancing by increasing Treg cell populations while suppressing Th17 responses and by enriching beneficial butyrate-producing microbiota (e.g., Prevotellaceae). This illustrates a synergistic mechanism in which both the drug and carrier degradation products actively participate in resolving intestinal inflammation [38]. Furthermore, coating liposomes with a Nutriose/chitosan complex can construct nanocapsules for delivering quercetin. This polymer coating not only achieves colon targeting via microbial enzyme degradation but, itself acting as a prebiotic, can also repair the damaged mucosal barrier, significantly enhancing the retention of quercetin in colonic tissue and its antioxidant efficacy [55].

**Figure 6:**
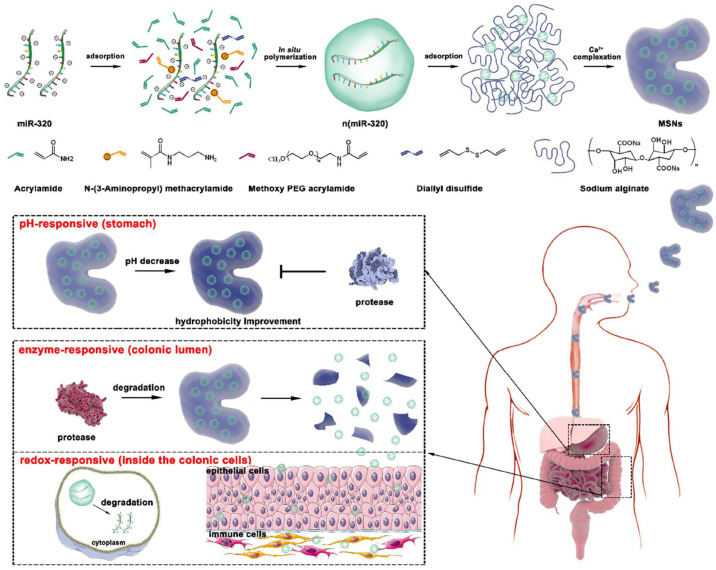
Schematic illustration of the hierarchical multi-stimuli-responsive delivery of nucleic acid therapeutics for IBD. (**Top**) Fabrication of alginate-based polymeric networks encapsulating disulfide-crosslinked nanocapsules. (**Bottom**) Cascade delivery process: (1) gastric protection via pH-induced alginate contraction; (2) colon-specific activation through enzyme-mediated matrix degradation and release of nanoscale carriers; and (3) intracellular release via glutathione-triggered cleavage of disulfide bonds. This sequential pH/enzyme/redox responsiveness enables efficient mucus penetration, cellular uptake, and cytosolic delivery of miR-320. Reprinted with permission from Reference [74]. Copyright American Chemical Society 2022.

However, the pathological environment of IBD is highly complex, and a single enzyme-responsive mechanism often struggles to overcome the multiple barriers of the gastrointestinal tract and achieve intracellular delivery. Consequently, multi-stage delivery systems integrating enzyme responsiveness with other stimuli (e.g., pH and redox conditions) have emerged. For example, a multi-stimuli-responsive polymeric composite assembled from alginate and disulfide bond-crosslinked polymeric nanocapsules demonstrates precise spatiotemporal delivery capability (Figure 6). A representative system is a multistage-responsive nanocomplex (MSN) platform delivering miR-320. This system employs a hierarchical “protection–release–penetration” cascade. Initially, MSNs (300–900 nm) are formed by complexing alginate with disulfide-crosslinked polymeric nanocapsules (24–36 nm). In the gastric environment (pH 1.2), the alginate shell protonates to form a protective barrier. Upon reaching the colon, microbiota-derived enzymes degrade the alginate shell, triggering a size-reduction process that releases the smaller nanocapsules. This size transition enables efficient penetration through the colonic mucus and facilitates access to deeper tissue layers. Following cellular uptake, intracellular glutathione cleaves the disulfide bonds, resulting in controlled release of miR-320. In addition, the system exhibits “drug–excipient synergy,” as alginate-derived products modulate the gut microbiota, while Ca^2+^ ions contribute to intestinal barrier repair. Collectively, the integration of pH, enzyme, and redox responsiveness, together with size transformation, enhances targeted therapeutic efficacy [74].

### 3.8. Comparative Evaluation of Different Polymeric DDS for IBD

Although enzyme-responsive strategies provide precise biological gating mechanisms, the overall therapeutic performance of these systems is fundamentally governed by carrier architecture. Polymeric delivery platforms, primarily classified as polymer prodrugs, nanocarriers (e.g., nanoparticles and micelles), and hydrogels/microgels, exhibit distinct structure–function relationships that critically influence drug loading, stability, tissue distribution, and release behavior. A systematic comparison of these architectures is therefore essential to guide rational design and clinical translation.

Polymer prodrugs enable exceptionally high and stoichiometrically defined drug loading through covalent conjugation, ensuring minimal premature leakage during gastrointestinal transit. However, their release kinetics are intrinsically dependent on enzymatic accessibility and local microenvironmental conditions, which may lead to incomplete or delayed drug activation [75]. Furthermore, the complexity of multi-step synthesis and the potential accumulation or toxicity of residual polymer fragments remain important translational considerations.

Polymeric nanocarriers, typically assembled through non-covalent interactions, offer superior versatility in encapsulating hydrophobic drugs and protecting them from degradation in the upper gastrointestinal tract. Their nanoscale dimensions facilitate deep penetration into inflamed tissues and efficient uptake by immune cells, enabling both extracellular and intracellular drug delivery [76]. Nevertheless, their structural stability is often compromised in the dynamic gastrointestinal environment, making them susceptible to premature burst release without additional protective strategies (e.g., enteric coatings). In addition, scalability and batch-to-batch reproducibility remain key challenges for clinical translation.

Hydrogels and microgels, in contrast, represent macroscopic or mesoscopic networks characterized by high water content and excellent biocompatibility. These systems are particularly advantageous for the delivery of hydrophilic drugs, biologics, and living therapeutics, owing to their protective matrix and tunable physicochemical properties. Their strong mucoadhesion prolongs residence time at inflamed sites, enhancing local drug exposure [77]. However, their relatively large size limits penetration into deeper mucosal layers, and their performance is highly sensitive to environmental factors such as pH, ionic strength, and fluid dynamics, which may vary significantly among patients with IBD.

Collectively, these distinctions highlight that no single platform is universally optimal; rather, the selection of an appropriate delivery architecture should be guided by drug properties, target site, and disease state. A detailed comparative summary is provided in Table 2.

## 4. Future Perspectives

The translational landscape of nanomedicine in IBD is currently at a critical, yet challenging, inflection point. While the highly sophisticated, multi-stimuli enzyme-responsive systems detailed in this review remain firmly entrenched in the preclinical stage, foundational micro- and nanomedicines have begun to enter human clinical trials, providing crucial preliminary proof of concept for nanoscale IBD therapy. For instance, SinaCurcumin^®^ and Theracurmin^®^ polymeric nano-micellar formulations of curcumin are currently undergoing Phase II/III clinical evaluation for mild-to-moderate UC [78]. These trials offer early clinical indications that polymeric nanocarriers (~100 nm) can potentially navigate the colonic mucus barrier to enhance the bioavailability of hydrophobic phytochemicals. Similarly, Ferumoxytol, a carbohydrate polymer-coated iron oxide nanoparticle (~30 nm) approved for IBD-associated anemia, is being evaluated in clinical imaging trials for its ability to specifically target macrophages in inflamed intestinal regions [79]. Furthermore, biological nanocarriers such as mesenchymal stem cell-derived exosomes (e.g., NCT04356300 for Crohn’s fistulas) are advancing through early Phase I/II trials [80,81]. While these milestones represent significant translational progress, it must be emphasized that these candidates are still navigating the arduous clinical trial pipeline and are far from becoming established standard-of-care therapeutics. Upgrading from these initial exploratory nanomedicines to next-generation “smart” enzyme-responsive systems presents even more formidable translational and regulatory hurdles.

### 4.1. Inter-Patient Heterogeneity

The performance of microbiota-dependent systems is strongly influenced by interindividual variability in gut microbiome composition and enzymatic activity. Severe dysbiosis may compromise carrier activation. Future development should integrate companion diagnostics, such as fecal enzyme profiling and microbiome sequencing, to enable personalized selection of delivery systems [82]. The incorporation of machine-learning-based predictive models may further enhance precision therapy.

### 4.2. Toward Logic-Gated Multi-Responsive Systems

Single-enzyme responsiveness may be insufficient in the complex gastrointestinal environment. Next-generation systems should incorporate multi-stimuli strategies operating under Boolean logic principles (e.g., simultaneous response to elevated pH and high MMP levels). Such logic-gated designs can minimize off-target release during transit while ensuring rapid activation at inflamed lesions [83].

### 4.3. Improving Translational Predictability

Current evidence largely relies on chemically induced colitis models, which do not fully replicate the chronic and microbiota-dependent features of human IBD. Advanced models including humanized microbiome systems, patient-derived intestinal organoids, and gut-on-a-chip platforms may provide more predictive evaluation of enzyme activity and drug release behavior.

### 4.4. Manufacturing and Regulatory Considerations

Despite the ongoing clinical evaluation of first-generation polymeric micelles and coated nanoparticles, the path to regulatory approval for advanced enzyme-responsive polymeric nanomedicines is obstructed by a complex constellation of manufacturing and regulatory bottlenecks. Primarily, this is due to the absence of globally harmonized regulatory frameworks. Agencies such as the FDA, EMA, and PMDA continue to exhibit significant heterogeneity in their definitions, characterization requirements, and approval pathways for “smart” nanoparticle-based therapeutics [84]. This regulatory uncertainty is profoundly exacerbated by the disease pathology itself: the inherent inter-patient variability in colonic microbiome composition and enzymatic activity in IBD patients leads to highly unpredictable in vivo drug release kinetics, complicating standardized safety and efficacy evaluations [85]. Adopting standardized roadmaps, such as the recently proposed DELIVER framework, will be a vital step toward integrating complex design principles with rigorous regulatory expectations.

Furthermore, the Chemistry, Manufacturing, and Controls (CMC) of these sophisticated responsive systems pose formidable technical challenges. Transitioning from laboratory-scale synthesis to scalable Good Manufacturing Practice (GMP) production requires maintaining strict batch-to-batch consistency regarding nanoparticle size, polydispersity, and surface functionalization. A particularly critical and often underappreciated challenge lies in establishing robust sterilization protocols. Conventional terminal sterilization methods (e.g., gamma irradiation, autoclaving) risk compromising the structural integrity of the delicate “chemical switches” (such as azo bonds, ester linkages, or enzyme-cleavable peptides) embedded within the polymer backbone [86]. Consequently, developing nondestructive sterilization techniques and establishing standardized in vitro release models that accurately simulate the highly heterogeneous human colonic enzymatic environment are urgently needed. Ultimately, bridging the cavernous gap between preclinical innovation and actual clinical practice will require sustained interdisciplinary collaboration to ensure the precision, reproducibility, and safety demanded for regulatory approval.

### 4.5. AI-Assisted Rational Design

Artificial intelligence (AI) offers a powerful approach to accelerate the rational design of enzyme-responsive polymeric drug delivery systems. Machine learning models can be trained on existing datasets to predict enzyme–substrate interactions, polymer degradation kinetics, and structure–property relationships, enabling more efficient identification of optimal materials. In particular, AI-assisted screening can guide the selection of enzyme-cleavable linkers and polymer architectures with desired stability and responsiveness under physiological conditions. In addition, data-driven modeling can help optimize key parameters such as particle size, surface chemistry, and release profiles, reducing reliance on trial-and-error experimentation. Coupled with high-throughput synthesis and characterization, AI-based strategies have the potential to significantly shorten development timelines and improve the translational efficiency of next-generation enzyme-responsive delivery systems.

### 4.6. Navigating the Trade-Off Between Mucoadhesion and Mucus Penetration

Although mucoadhesion has been widely exploited to enhance the retention of polymeric carriers at inflamed sites, a closer examination of the colonic mucosal barrier reveals an inherent physiological paradox [87]. In inflamed tissues, the mucus layer is not static but highly dynamic, characterized by continuous secretion and rapid turnover. Consequently, carriers exhibiting strong non-specific adhesion, particularly via electrostatic interactions, may become immobilized within the loosely structured outer mucus layer and subsequently eliminated through mucus shedding before reaching the epithelial interface. This highlights a critical trade-off between mucoadhesion and mucus penetration. From a design perspective, optimal carriers should initially exhibit mucus-penetrating characteristics, such as near-neutral surface charge, hydrophilic and densely grafted polymer shells (e.g., PEG), or enzyme-triggered size reduction, enabling rapid diffusion through the mucin network. Following penetration, the exposure of adhesive ligands or localized payload release at the epithelial surface can maximize drug bioavailability. Therefore, the development of dynamically switchable systems capable of transitioning from a mucus-penetrating to a cell-adhesive state in response to disease-specific enzymatic cues represents a promising strategy for overcoming mucosal barriers and enhancing therapeutic efficacy. This paradigm shift from static adhesion to adaptive interface engineering is likely to play a key role in the next generation of enzyme-responsive nanomedicines.

## 5. Conclusions

Enzyme-responsive polymeric drug delivery systems have redefined localized therapeutic strategies for IBD by exploiting the disease-specific enzymatic microenvironment, including both host-derived inflammatory enzymes and microbiota-associated enzymes. This intrinsic biological triggering mechanism enables site-specific drug activation and spatiotemporally controlled release, thereby enhancing drug accumulation at inflamed lesions while minimizing premature release during gastrointestinal transit. Importantly, recent advances demonstrate that polymers should no longer be viewed as inert carriers. The concept of “drug–excipient synergy” highlights that bioactive degradation products, particularly from natural polysaccharides and polymer prodrugs, can actively contribute to therapeutic efficacy by modulating immune responses, promoting mucosal repair, and restoring microbiota homeostasis. From a design perspective, future systems should move beyond single-trigger strategies toward multi-stage, multi-stimuli-responsive architectures capable of overcoming sequential biological barriers. In particular, dynamic features such as enzyme-triggered size reduction and surface charge modulation are critical for balancing gastrointestinal stability with effective mucus penetration and retention.

Despite promising progress, the clinical translation of enzyme-responsive systems remains challenging. Key barriers include inter-patient variability in microbiota composition and enzymatic activity, limited predictive preclinical models, and significant manufacturing and regulatory constraints. Addressing these challenges will require the integration of advanced evaluation platforms, standardized regulatory frameworks, and scalable production strategies. Looking forward, emerging approaches such as logic-gated multi-responsive systems, AI-assisted material design, and personalized delivery strategies based on microbiome profiling are expected to further advance the field. Collectively, the integration of bioactive material selection, adaptive carrier design, and translational considerations provides a comprehensive framework for the development of next-generation enzyme-responsive nanomedicines for IBD therapy.

## Figures and Tables

**Table 1:** Overview of enzyme-responsive DDSs in IBD: enzymatic sources, localization, and material design strategies.

Enzyme Trigger	Delivery System Design	Key Materials & Linkers	Drug Payload	Therapeutic Outcomes (Preclinical Models)	Ref.
**Esterase**	Multifunctional hydrogel beads	Ascorbyl palmitate/Sodium alginate (ester linkages)	Apigenin & Butyrate (prodrug)	Inhibits NF-κB via CK2/p65; shifts metabolism to β-oxidation; modulates microbiota (↑ *Bacteroidetes*); repairs intestinal barrier.	[39]
	Core–shell lipid nanoparticles	Chitosan shell/Qu-SS-Gcc lipid core (ester bonds)	Dexamethasone & Quercetin	Protects epithelial Caco-2 cells; upregulates E-cadherin; reduces TNF-α, IL-6, NO in macrophages; targets colon actively.	[40]
	Nanoliposomes (biomucoadhesive)	Chitosan coated DMPG/β-sitosterol−sinapic acid conjugate	MNS (NLRP3 inhibitor) & Sinapic acid	Prolonged 48 h retention; downregulates NLRP3, Caspase-1, IL-1β; upregulates MUC5AC; reduces neutrophil infiltration.	[30]
**Azoreductase**	Stimuli-responsive hydrogel	2-hydroxyethyl methacrylate/methacrylic acid (azobenzyl crosslinker)	Metronidazole & Mesalamine	pH-dependent swelling limits gastric release; triggers targeted burst release in colonic caecal matter; enhances dissolution.	[41]
	Dual-sensitive (pH/enzyme) nanoparticles	Eudragit S100 shell/Azo-polyurethane core	Coumarin-6 (Model)	Prevents burst release at pH 7.4; shows 5.5-fold higher accumulation in the inflamed colon; selectively targets lesions.	[42]
	Mucoadhesive nano-micelles	PEG-Azo-PLGA/Catechol-modified TPGS	Curcumin	Catechol induces strong mucoadhesion; downregulates TLR4/MyD88/NF-κB pathway; mitigates colitis and regulates flora.	[43]
**Hyaluronidase**	Self-assembled nanoparticles	Amphiphilic hyaluronic acid (HA) conjugates	Budesonide	Actively targets CD44-overexpressing inflamed cells; significantly decreases IL-8 and TNF-α secretion with excellent biocompatibility.	[44]
	Dual-targeting core–shell nanoparticles	Calcium pectinate shell/HA-modified Lactoferrin core	Rhein	Targets epithelial cells & macrophages; resists gastric digestion; inhibits TLR4/MyD88/NF-κB pathway; accelerates colonic healing.	[45]
	Theranostic core–shell nanoprobes	High-molecular-weight HA/Cerium oxide (CeO2)	Curcumin & CeO2	Enables CT imaging-guided tracking; synergistically scavenges ROS; exerts robust anti-inflammatory and antioxidant effects.	[26]
	Self-assembled nanomedicine	Hyaluronic acid-bilirubin (HA-BR) conjugates	Bilirubin (scavenger)	Protects epithelium against apoptosis; markedly increases beneficial flora (*Akkermansia muciniphila*, *Clostridium XIVα*).	[46]
**Cellulase**	Layer-by-Layer (LbL) solid lipid nanoparticles	Sodium cellulose sulfate (NaCS)/Chitosan polyelectrolytes	Budesonide	Anchored cellulase-responsive layers control specific colonic release; exhibits excellent anti-inflammatory activity in DSS mice.	[31]
	Dual pH/electro-responsive hybrid hydrogels	Bacterial cellulose nanofibers/Sodium alginate	Ibuprofen	Controlled Fickian diffusion mediated by pH and external electric field; highly swellable in alkaline/colonic conditions.	[47]
	Nanofiber-boosted microparticles	Retrograded starch/pectin/Cellulose nanofibers (CNF)	5-ASA	CNF enhances mucoadhesion (up to 3.4 N); increases intestinal permeability tenfold; reduces LPS-induced inflammation in vivo.	[48]
**α-Amylase**	ROS-scavenging nanoparticles	Hydroxyethyl starch (HES)-curcumin conjugates	Dexamethasone & Curcumin	Degraded by overexpressed α-amylase; scavenges ROS; internalized by macrophages; improves combination therapy efficacy.	[49]
	Enzyme/ROS dual-sensitive nanoplatform	β-cyclodextrin/4-(hydroxymethyl) phenylboronic acid	Celastrol	On-demand release triggered by ROS and enzymes; promotes macrophage polarization; rebalances microbiota; recovers barrier.	[50]
	Nanoparticle-in-microparticle system	Curcumin-cyclodextrin core/Chitosan & unsaturated alginate shell	Curcumin	Rapid macrophage uptake; promotes epithelial barrier integrity; reshapes gut microbiota; strong colonic biodistribution.	[32]
	Starch film-coated microparticles	High-amylose cornstarch (RS2)/Retrograded starch (RS3) film	5-ASA	Highly resistant to upper GIT digestion; provides accurately targeted bioactive compound delivery to the colonic lumen.	[51]
	Microspheres (combination therapy)	Hydroxyethyl starch-curcumin conjugates	Dexamethasone & Curcumin	Achieves exceptionally high drug loading; mitigates spleen enlargement; alleviates oxidative stress and modulates gut flora.	[52]
**MMPs**	Inflammation-targeting hydrogel microfibers	Ascorbyl palmitate (GRAS amphiphile)	Dexamethasone	Preferentially adheres to inflamed epithelial surfaces/lesions; lowers systemic drug exposure; limits off-target toxicity.	[53]
	Dynamic multi-arm hydrogels	Gelatin/Multi-arm PEG (dynamic hydrazone bonds)	5-ASA	Tunable colonic retention (12–36 h); consumes MMP-9; reduces collagen deposition; facilitates robust colon tissue repair.	[54]
**Others (Pectinase/Inulinase)**	Coated nanovesicles	Hydrogenated soy phosphatidylcholine/Chitosan & Nutriose	Quercetin	Prebiotic effects of chitosan/nutriose synergize with antioxidant quercetin; ameliorates TNBS-induced colitis symptoms.	[55]
	Core–shell self-assembled nanostructures	Inulin-dehydropeptide conjugates (ester linkage)	Ornidazole	Degraded specifically by colonic inulinase; peptide conjugation ensures stability against broad pH and upper GIT proteases.	[56]
	Prebiotic core–shell nanoparticles	Pectin/Chitosan shell/PLGA core	Sulfasalazine	Pectinase-triggered degradation yields prebiotic oligosaccharides; explicitly restores Treg/Th17 immune cell balance.	[38]

**Table 2:** Comparative evaluation of enzyme-responsive polymeric delivery architectures for IBD therapy.

Architecture Type	Advantages	Disadvantages	Optimal Payloads	Ref.
Polymer Prodrugs	Extremely high and predictable drug loading capacity. Minimal premature drug leakage in stomach/small intestine. Synchronized carrier degradation and drug release.	Complex, multi-step chemical synthesis. Release kinetics heavily dependent on enzymatic accessibility (steric hindrance). Potential toxicity of polymer backbones post cleavage.	Low molecular weight chemical drugs (e.g., 5-ASA, steroids)	[59,75]
Nanocarriers (Nanoparticles, Micelles, Nanocapsules)	High encapsulation efficiency for hydrophobic drugs. Nanoscale size enables deep penetration into inflamed submucosa. Easily internalized by immune cells (e.g., macrophages) for intracellular delivery. Easy to functionalize with targeting ligands.	Prone to premature burst release if not adequately shielded (e.g., requires enteric coating). Lower overall drug loading compared to prodrugs. Scale-up and batch to batch consistency challenges in self-assembly.	Hydrophobic anti-inflammatory drugs (e.g., curcumin, dexamethasone)	[16,43]
Hydrogels & Microgels	Excellent biocompatibility and structural protection for delicate cargos. Strong mucoadhesive properties prolong mucosal residence time. Highly tunable swelling and degradation kinetics. Ability to deliver a diverse range of payloads concurrently.	Bulky size restricts deep tissue penetration (action is primarily luminal/surface). Slower drug release rates depending on network crosslinking density. Swelling behavior may be altered by individual variations in gut fluid volume/pH.	Hydrophilic drugs, biologics (proteins, antibodies), live probiotics	[39,53,54]

## Data Availability

Not applicable.
